# Prognostic and functional implications of left atrial late gadolinium enhancement cardiovascular magnetic resonance

**DOI:** 10.1186/s12968-018-0514-3

**Published:** 2019-01-03

**Authors:** Michael Quail, Karl Grunseich, Lauren A. Baldassarre, Hamid Mojibian, Mark A. Marieb, Daniel Cornfeld, Aaron Soufer, Albert J. Sinusas, Dana C. Peters

**Affiliations:** 1 Department of Internal Medicine (Cardiology), Yale School of Medicine, 300 Cedar St, New Haven, CT 06520 USA; 2 Department of Radiology and Biomedical Imaging, Yale School of Medicine, New Haven, CT USA; 3 Centre for Cardiovascular Imaging, Institute of Cardiovascular Science, University College London, London, UK

**Keywords:** Atrial fibrillation, Left atrium, Ejection fraction, Late gadolinium enhancement, Cardiovascular magnetic resonance

## Abstract

**Background:**

Left atrial (LA) late gadolinium enhancement (LGE) on cardiovascular magnetic resonance (CMR) imaging is indicative of fibrosis, and has been correlated with reduced LA function, increased LA volume, and poor procedural outcomes in cohorts with atrial fibrillation (AF). However, the role of LGE as a prognostic biomarker for arrhythmia in cardiac disease has not been examined.

**Methods:**

In this study, we assessed LA LGE using a 3D LGE CMR sequence to examine its relationships with new onset atrial arrhythmia, and LA and left ventricular (LV) mechanical function.

**Results:**

LA LGE images were acquired in 111 patients undergoing CMR imaging, including 66 patients with no prior history of an atrial arrhythmia. During the median follow-up of 2.7 years (interquartile range (IQR) 1.8–3.7 years), 15/66 (23%) of patients developed a new atrial arrhythmia. LA LGE ≥10% of LA myocardial volume was significantly associated with an increased rate of new-onset atrial arrhythmia, with a hazard ratio of 3.16 (95% CI 1.14–8.72), *p* = 0.026. There were significant relationships between LA LGE and both LA ejection fraction (*r* = − 0.39, *p* < 0.0005) and echocardiographic LV septal e’ (*r* = − 0.24, *p* = 0.04) and septal E/e’ (*r* = 0.31, *p* = 0.007).

**Conclusions:**

Elevated LA LGE is associated with reduced LA function and reduced LV diastolic function. LA LGE is associated with new onset atrial arrhythmia during follow-up.

**Electronic supplementary material:**

The online version of this article (10.1186/s12968-018-0514-3) contains supplementary material, which is available to authorized users.

## Introduction

Late gadolinium enhancement (LGE) on cardiovascular magnetic resonance (CMR) has been used to characterize areas of fibrosis in the left atrium (LA) in patients with atrial fibrillation (AF) [1]. Pre-ablation areas of LA LGE have been associated with decreased atrial function [2, 3] and lower voltage on collocated endocardial voltage maps [4, 5]. Additionally, greater LA LGE has been found to be associated with heightened stroke risk, [6] cardiovascular events, [7] time in AF, pattern of AF [8], and failure of AF therapies [9, 10]. The reproducibility of LA LGE measurement has been demonstrated in several recent studies [11, 12], and LGE sequences are commonly available on most scanners [13].

Previous epidemiological studies have described a variety of clinical risk factors for AF. Associations have been found with age, LA size, valvular disease, heart failure, hypertrophic cardiomyopathy, and recently LA strain by echocardiography and CMR [14–18]. LA LGE has not been examined as a risk factor for new onset atrial arrhythmia. In part this is because, despite the value of LA LGE in patients already found to have AF, patterns of LA LGE in patients without atrial arrhythmia have remained largely unexamined until recently [19].

The pathophysiological factors underlying the development and maintenance of AF are incompletely understood. It is currently thought to occur via an interaction between impulses generated by aberrant tissue in the region of the pulmonary veins and a remodeled, enlarged, fibrotic, and electrically susceptible atrial substrate [20, 21]. Atrial remodeling (i.e. fibrosis and enlargement) occurs in a range of pathological settings including mitral valve disease, abnormal diastolic function (heart-failure with preserved ejection fraction, HFpEF) and myocardial ischaemia [22]. LA LGE is a noninvasive measurement of atrial fibrosis and remodeling; however an association between LA LGE and the risk of developing atrial arrhythmia has not been described.

The purpose of this study was to characterize the presence of LA LGE in a heterogeneous population undergoing CMR to assess its relationship with new onset atrial arrhythmia. Furthermore, we sought to assess the functional relationship between LA LGE and indices of LA and left ventricular (LV) function, including diastolic function.

## Methods

### Patient population and clinical data

This study was an institutional review board (IRB) approved, retrospective review of all subjects imaged with a 3D LGE CMR sequence at our institution from 2012 to 2014. LGE atrial imaging was added to the standard clinical CMR protocol for patients undergoing myocardial assessment for various indications.

Exclusion criteria for the study included: 1. Unsatisfactory atrial LGE imaging quality, 2. Inadequate clinical record (without documented follow-up), 3. Age < 20 years, or 4. Prior cardiac surgery or prior ablation.

New atrial arrhythmia during follow-up was determined from review of the electronic medical records of electrophysiological investigations. Atrial arrhythmias included new, permanent or paroxysmal AF, atrial tachycardia or atrial flutter. The presence or absence of new arrhythmia was identified on devices (implanted cardiodefibrillator (ICD), permanent pacemaker (PPM) or implanted event monitor) or Holter monitor (*n* = 42). For patients without an implanted device or Holter, a negative finding was considered to have occurred if there was no history of palpitations and a 12 lead electrocardiogram (ECG) with an atrial arrhythmia at follow-up (*n* = 24). Echocardiographic measurements of LV tissue Doppler and mitral valve pulse wave Doppler, when available, were recorded to assess the relationship of LV diastolic dysfunction with LA LGE and atrial function.

### LV and LA CMR imaging: 2D cine and 3D late gadolinium enhancement

CMR was performed on Siemens 1.5 T scanner (Aera, Siemens Healthineers, Erlangen Germany). Subjects were imaged with cine CMR in two chamber and four chamber views, as well as with a short axis LV stack from the base to apex. Scan parameters include: balanced steady state free precession cine with retrospective ECG-gating, TR (repetition time)/TE (echo time)/θ = 3 ms/1.5 ms/60°, 30 cardiac phases, and 1.4 × 1.4 × 8 mm^3^ resolution. The LA LGE sequenced was performed during mid-ventricular diastole using an ECG-triggered and navigator-gated, fat-saturated 3D gradient echo inversion recovery sequence, 15–25 min after administration of 0.2 mmol/kg gadolinium contrast agent (Gadobutrol, Bayer Healthcare, Leverkusen, Germany). Voxel size was 1.3 × 1.3 × 3.0 mm^3^ with interpolation to 0.7 × 0.7 × 1.5 mm^3^. Additional scan parameters were: TR/TE/θ = 5.3 ms/2.1 ms/15°. Twenty-seven views per segment were acquired, in a ky-centric order. A parallel imaging acceleration factor of 2 was used.

### Image analysis

**Fig. 1:**
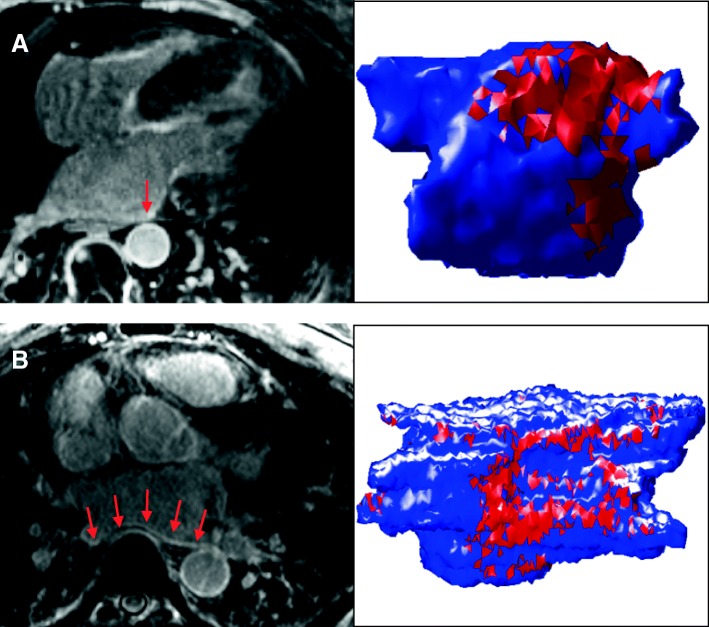
3D late gadolinium enhancement (LGE) slices and 3D segmentation of atrial LGE in two representative patients developing a new atrial arrhythmia. **a**) 52 year old man with hypertrophic cardiomyopathy, who developed atrial flutter 3.3 years after CMR. **b**) A 65 year old man with coronary artery disease, who developed atrial fibrillation (AF) 7 months after CMR. Red arrows point to regions of LGE enhancement (bright signal)

Quantitative segmentation of the LA LGE volume was performed manually using open-source 3D SLICER (4.3.1, NA-MIC), using a patient-specific threshold determined by the contrast to noise ratio (CNR) of normally enhanced tissues in the field of view, i.e. the enhanced valves, as previously described [23]. Enhancement of extra-atrial tissues and artifacts was manually removed. All LGE analysis was conducted by a single CMR expert (DCP) who was blinded to clinical data. The volume of segmented LGE enhancement was obtained using summation of segmented areas of enhancement in each axial slice (Fig. 1). For inter and intra-observer variability, the LA LGE analysis was performed and repeated a week later by the same reader, and by a second reader, blinded to any other data.

**Fig. 2:**
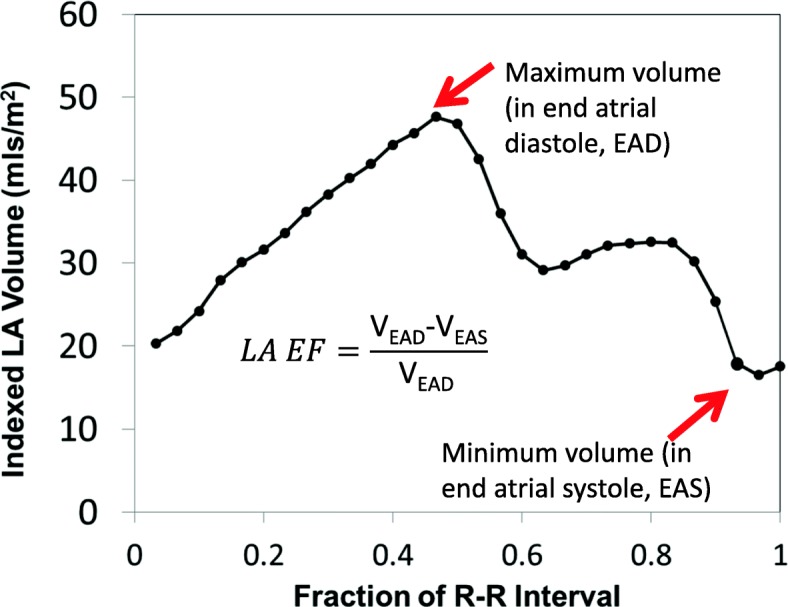
Measurement of left atrial (LA) volumes and ejection fractions (EF). Representative curve of LA volume during the cardiac cycle with formulas used to quantify atrial functions. The times of atrial end-diastole (with maximum volume) and atrial end-systole, with minimum volume, are indicated

Measurements of the LA end-diastolic volume and LA end-systolic volume were approximated by biplane area-length method [24] and used to quantitate LA ejection fraction (EF), using CMR42 (Circle Cardiovascular Imaging, Calgary, Alberta, Canada). LA end-diastolic and end-systolic volumes were measured at *atrial* end-diastole, and *atrial* end-systole, respectively (Fig. 2); these are referred to as maximum and minimum volumes. Volumes were indexed to body surface area (BSA). An extension of this method was used to estimate the total volume of the LA wall at end-diastole, with an assumed average LA wall thickness of 2.1 mm [25] for all subjects and the surface area of the LA modeled as a scalene ellipsoid. LA LGE volume as quantitated above was divided by total LA tissue volume to calculate an LA LGE percentage.

### CHARGE-AF risk scores

The CHARGE-AF risk score was calculated using the multivariable regression model proposed by Alonso et al. [26]. The following data was used for this score: age, race, height, weight, systolic blood pressure, diastolic blood pressure, current smoking status, anti-hypertensive medication, diabetes, heart failure, and prior myocardial infarction.

### Statistics

STATA 13.1 (Stata Corporation, College Station, Texas, USA) was used for statistical analysis. Descriptive statistics are expressed as mean (±95% confidence interval) when normally distributed, and geometric mean (±95% confidence interval of geometric mean) when non-normally distributed, unless specified. Proportions are expressed as percentages. In describing the relationship between LA LGE with clinical and cardiac variables, LGE was analyzed as a percentage of estimated LA tissue volume. Data were examined for normality using the Shapiro-Wilk test, and where appropriate, non-normally distributed variables were transformed prior to analysis. Based on this analysis, LA LGE, LA EF, and LA volumes were transformed using the square-root operation, as noted in the tables.

The independent samples t-test was used to compare differences in normally distributed data between groups; Welch’s correction was employed for unequal variances. Proportions test was used to compare proportions amongst groups.

We used logistic regression analysis to assess the relationship between new onset atrial arrhythmia and the following clinical parameters: age, sex, BMI, hypertension; and the following functional parameters: LA LGE, minimum and maximum LA volume index, LA EF, LV EF and presence of LV LGE. Multivariable logistic regression analysis was used to assess independent relationships between new-onset atrial arrhythmia and associated covariates. Covariates with a *p* < 0.1 were eligible for inclusion in the multivariable model. Cox regression analysis was used for time to event analysis. Variables transformed for normality were converted to z-scores for use in regression analysis. The area under the receiver operating characteristics curve was used to identify the degree of LA LGE with the greatest classification accuracy.

Pearson’s correlation coefficient was used to analyse simple linear relationships between LA LGE and functional variables from echocardiography and CMR. Intraclass correlation coefficient (ICC) and Bland-Altman analysis was used to assess inter and intra-observer variability.

The level of alpha considered for statistical significance was 0.05.

## Results

### Demographics

Out of 229 subjects initially eligible for this study, 93 (40%) were excluded due to poor image quality (e.g. excessive motion artefact, poor contrast, or poor inversion time choice), resulting in 136 subjects. An additional 25 subjects did not have adequate clinical follow-up, yielding 111 subjects for analysis. Sixty six (59%) subjects were male and the participant mean age was 53.2 years (95% CI 50.6–55.7) (Table 1). The diagnosis and CMR indications are noted in Table 2.

**Table 1:** Demographic and clinical data

	Mean (95% CI) Or n (%)
Age (years)	53.2 (50.7–55.7)
BMI (kg/m^2^)	28.2 (27.2–29.3)
Male	66 (59%)
Prior Atrial Arrhythmia	45 (41%)
Atherosclerosis Risk Factors	
Tobacco use	39 (38%)
Hyperlipidemia	53 (47%)
Hypertension	56 (53%)
Diabetes	12 (11%)
Any LV LGE	28 (25%)
CMR Metrics	
LA LGE (%)^a^	4.6 (0–36.1)
LA Volume minimum (ml/m^2^)^b^	24.2 (21.9–26.7)
LA Volume maximum (ml/m^2^)^b^	46.1 (43.1–49.3)
LV EDV index (ml/m^2^)^b^	85.6 (81.3–90.1)
LA EF (%)^b^	38.1 (31.7–45.9)
LV EF (%)^b^	48.9 (46.3–51.6)

*BMI* body mass index, *CMR* cardiovascular magnetic resonance, *EDV* end-diastolic volume, *EF* ejection fraction, *LA* left atrium/left atrial, *LV* left ventricle/left ventricular

^a^Median (Range), ^b^Geometric Mean (95% CI)

A large minority (41%) of subjects had a history of atrial arrhythmia at the time of imaging (Table 2). As expected patients had a significant burden of conventional cardiovascular disease risk factors (Table 1). No subjects had an ICD or PPM at the time of CMR imaging.

**Table 2:** Clinical diagnosis at time of imaging

Diagnosis	Total *n* = 111	No prior Atrial Arrhythmia *n* = 66	Prior Atrial Arrhythmia *n* = 45
Dilated cardiomyopathy	36 (32.4%)	22 (33.3%)	14 (31.1%)
Pre-AF ablation	25 (22.5%)	–	25 (55.6%)
Ventricular arrhythmia	14 (12.6%)	12 (18.2%)	2 (4.4%)
Hypertrophic cardiomyopathy	8 (7.2%)	7 (3.0%)	1 (2.2%)
Sarcoid	8 (7.2%)	8 (12.1%)	–
ARVC	6 (5.4%)	6 (9.1%)	–
Normal/Family history of cardiomyopathy	5 (4.5%)	5 (7.6%)	–
Coronary artery disease	4 (3.6%)	4 (6.1%)	–
Valvular disease	3 (2.7%)	2 (3.0)	1 (2.2%)
Pericarditis	2 (1.8%)	–	2 (4.4%)

*ARVC* arrhythmogenic right ventricle cardiomyopathy

The median duration of clinical follow-up was 3.4 years (interquartile range (IQR) 2.6–4.2 years) during which 5/111 (4.5%) patients died. A cardiac cause of death was recorded in 3 patients (acute heart failure, death after biventricular assist device implantation & cardiac transplantation, and severe hypertrophic cardiomyopathy), one patient died of respiratory failure secondary to lung carcinoma, and the cause of death was unavailable in one patient.

### LA LGE reproducibility, associations and group differences

LA LGE segmentation reproducibility was performed in a subset of 24 patients. Intra-observer variability was excellent (bias ±2SDs = − 0.3% ± 2.9%, ICC = 0.94); inter-observer variability was moderate to good (bias ±2SDs = 0.6% ± 7.3%, ICC = 0.71). See Additional file 1: Table S1 and Figure S1 and S2. Mean LA LGE segmentation time was 5 ± 2 min for the first reader.

The median LA LGE was 4.6% (range 0–36.1%) with no differences in between male and female subjects (*p* = 0.7). The degree of LA LGE was significantly associated with age (*r* = 0.33, *p* = 0.001). Subjects with a history of hypertrophic cardiomyopathy had higher LA LGE compared to other participants (8.7% vs 3.6%, *p* = 0.02). LA LGE was significantly higher 12.9% (8.4–19.9%) in those who died compared to survivors 3.7% (2.9–4.7%), *p* = 0.008.

### New atrial arrhythmia

**Fig. 3:**
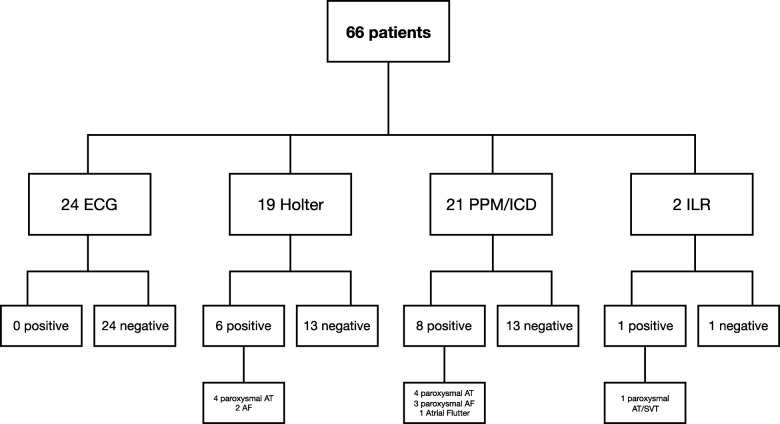
Flow diagram showing patients at risk of atrial arrhythmia, mode of diagnosis and atrial arrhythmia. AT = atrial tachycardia; ECG = electrocardiogram; ICD = implanted cardiodefibrillator; PPM = permanent pacemaker; SVT=supraventricular tachycardia

Sixty-six patients did not have a history of atrial arrhythmia at the time of CMR imaging. Figure 3 details the modes of diagnosing any new atrial arrhythmia in these patients. During a median follow-up of 2.7 years (IQR 1.8–3.7 years), 15 patients had evidence of a new atrial arrhythmia, including 5 patients developing AF, 9 patients developing an atrial tachycardia and one patient developing atrial flutter.

The degree of LA LGE was significantly higher in patients with new atrial arrhythmia compared to those without (6.8, 95% CI 4.2–11.1% vs 3.4, 95% CI 2.4–4.8%; *p* = 0.02). Using receiver operating characteristics analysis, a cutpoint of 10% LA LGE was identified as having the highest correct classification, 76% (area under curve (AUC), 0.68, *p* = 0.013) for new onset atrial arrhythmia.

**Fig. 4:**
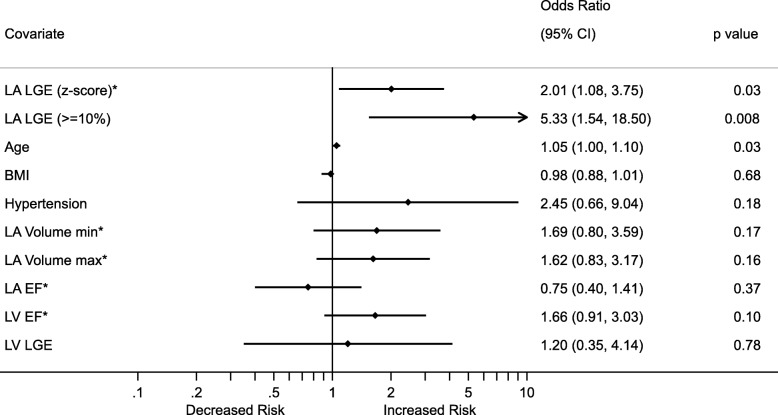
Forest plot of univariable odds ratios (OR) for risk of developing new atrial arrhythmia. *Data untransformed (age, body mass index (BMI)) or transformed (indexed LA volume, left ventricular (LV) EF, LA LGE) for normality and OR is expressed as the increase in odds of new atrial arrhythmia for 1 standard deviation increase in the normally transformed variable

Using logistic regression, the degree of LA LGE, age and LV EF were significantly associated with new onset atrial arrhythmia on univariable analysis. Considered as a continuous variable, a z-score (standard deviation) change in LA LGE was associated with an odds ratio (OR) 2.01 (1.08–3.75) for new atrial arrhythmia *p* = 0.03 (Fig. 4). The OR of atrial arrhythmia for LA LGE > =10% was 5.33 (1.54–18.50), *p* = 0.008). Multivariable regression analysis demonstrated a significant relationship between the development of new atrial arrhythmia and both LA LGE (continuous or binary) and LVEF (OR 2.20 [1.13–4.23], p = 0.02) (Table 3).

**Table 3:** Univariable and multivariable logistic regression analysis for new onset atrial arrhythmia in 66 patients without prior atrial arrhythmia

Parameters	Univariable Analysis		Multivariable Analysis	
	OR	Significance	OR	Significance
LA LGE^a^	2.01 (1.08–3.75)	***p*** **= 0.03**	^b^	
LA LGE (> = 10%)	5.33 (1.54–18.5)	***p*** **= 0.008**	6.94 (1.57–30.6)	***p*** **= 0.01**
Age (year)	1.05 (1.00–1.09)	***p*** **= 0.03**	1.04 (0.99–1.10)	*p* = 0.07
BMI (kg/m^2^)	0.98 (0.88–1.01)	*p* = 0.68	–	–
Hypertension	2.45 (0.66–9.04)	*p* = 0.18	–	–
LA Volume index minimum^a^	1.69 (0.79–3.59)	*p* = 0.17	–	–
LA Volume index maximum^a^	1.62 (0.83–3.17)	*p* = 0.16	–	–
LA EF^a^	0.75 (0.40–1.41)	*p* = 0.37	–	–
LV EF^a^	1.66 (0.91–3.03)	***p*** = 0.10*	2.20 (1.13–4.23)	***p*** **= 0.02**
Any LV LGE	1.2 (0.35–4.14)	*p* = 0.78	–	–

Covariates with univariate *p* < =0.1 were analysed using multivariable regression. ^a^Transformed for normality by square-root and Odd’s Ratio (OR) is expressed as the increase in odds of new atrial arrhythmia for 1 standard deviation increase in the normally transformed variable. ^b^OR 1.9 (1.01–3.50), *p* = 0.046 in multivariable logistic regression with LVEF. *Univariable factors with significance *p*<0.1 were included in multivariate analysis

**Fig. 5:**
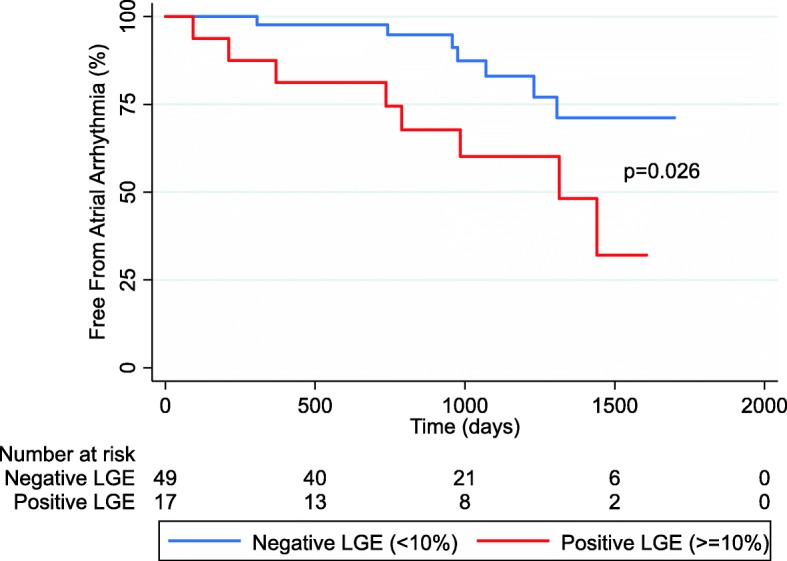
Kaplan Meier survival curve showing freedom from atrial arrhythmia from time of LA LGE assessment, stratified by positive (> = 10%) and negative (< 10%) LA LGE

Cox analysis demonstrated that patients with an LA LGE > =10% developed atrial tachyarrhythmia at a significantly increased rate than patients with less than 10%, hazard ratio 3.16 (1.14–8.72), *p* = 0.026, (Fig. 5).

To exclude ascertainment bias arising as a consequence of utilizing a negative 12 lead ECG and an absence of symptoms for some negative events, a sensitivity analysis was performed using only patients with implanted devices or Holter data (*n* = 42). In this analysis, the relationship between LA LGE > =10% and new atrial arrhythmia remained significant (Hazard ratio 3.4 [1.2–9.5], *p* = 0.017).

### CHARGE-AF risk score

CHARGE-AF scores could be calculated from available data in 32/66 (48%) of patients at risk of developing new atrial arrhythmia. There was no significant correlation between LA LGE and CHARGE-AF (*r* = 0.26, *p* = 0.15). Multivariable logistic regression analysis showed that both LA LGE positivity and CHARGE-AF score were independently associated with new onset atrial arrhythmia. The OR of atrial arrhythmia for LA LGE > =10% was 6.74 (1.01–44.8), *p* = 0.049 and for CHARGE-AF OR 1.79 (1.02–3.13), *p* = 0.043.

### Relationship of LA LGE with indices of LV and LA function

**Fig. 6:**
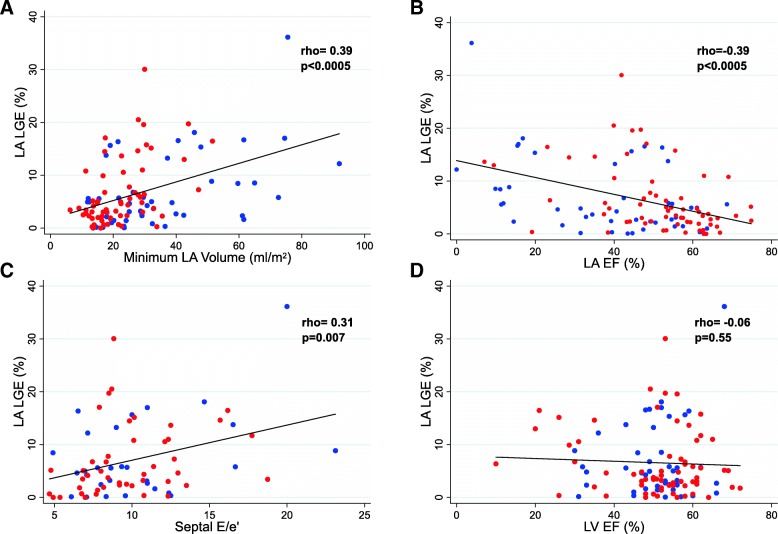
Relationship between LA LGE (%) and indexed minimum LA Volume (**a**), LA EF (**b**), echo tissue Doppler Septal E/e’(**c**) and LV EF (**d**). Red dots: No prior atrial arrhythmia at time of CMR, Blue dots: Prior atrial arrhythmia

The relationships between LA LGE and conventional metrics of LA and LV function are shown in Table 4 and Fig. 6. More extensive LA LGE was associated with worsening diastolic function but not LV systolic function (*p* = 0.55). LA LGE correlated with larger LA volumes (minimum volume *r* = 0.39, *p* < 0.001, maximum volume *r* = 0.24, *p* = 0.01) and lower LA ejection fraction (*r* = − 0.39, *p* < 0.001). These relationships were maintained following adjustment for age in multivariable regression.

**Table 4:** Relationship of CMR and Echo variables with LA LGE (%), transformed for normality by square-root

	All Subjects		No Prior Atrial Arrhythmia		Prior Arrhythmia	
	r	Significance	r	Significance	r	Significance
LA EF	**−0.39**	***p*** **< 0.0005**	**−0.45**	***p*** **< 0.0005**	**−0.37**	***p = 0.01***
LA Volume Index (minimum)	**0.39**	***p*** **< 0.0005**	**0.47**	***p*** **< 0.0005**	**0.39**	***p = 0.01***
LA Volume Index (maximum)	**0.24**	***p*** **= 0.01**	0.23	*p* =0.06	0.26	*p* = 0.08
LV EDV Index	0.07	*p* = 0.46	0.19	*p* =0.14	−0.14	*p* = 0.35
LV EF	−0.08	*p* = 0.38	−0.06	*p* =0.62	−0.04	*p* = 0.80
e’ Septal	**−0.24**	***p*** **= 0.04**	−0.28	*p* =0.07	−0.31	*p* = 0.10
e’ Lateral	−0.13	*p* = 0.25	−0.23	*p* = 0.11	0.06	*p* = 0.75
E/e’ Septal	**0.31**	***p*** **= 0.007**	**0.37**	***p = 0.01***	0.27	*p* = 0.16
E/e’ Lateral	0.13	*p* = 0.23	0.23	*p* = 0.10	0.02	*p* = 0.93
E/A Ratio	0.06	*p* = 0.63	0.04	*p* = 0.80	0.14	*p* = 0.52

All bold entries have significance *P* < 0.05

Patients without prior atrial arrhythmia had higher LA EF (51% vs 38%, *p* < 0.001) and the relationship between LA LGE and LA size and function was stronger in this group (4).

Seventy-eight patients (52/66 without prior atrial arrhythmia) had contemporaneous echocardiography data available for analysis (median interval between echo and CMR 61 days, IQR: 17-215 days). There were significant relationships between metrics of LV diastolic function and LA LGE: septal LV tissue Doppler e’ (*r* = − 0.24, *p* = 0.04) and septal E/e’ (*r* = 0.31, *p* = 0.007) (Fig. 6). There was no statistically significant relationship with LV lateral wall tissue Doppler metrics or mitral E/A ratio. LA EF correlated significantly with septal e’ (*r* = 0.26, *p* = 0.03), lateral e’ (*r* = 0.32, *p* = 0.006), and both septal E/e’ (*r* = − 0.27, *p* = 0.02) and lateral E/e’ (r = 0.32, *p* = 0.007).

## Discussion

The main finding of this study is that LA LGE, indicative of LA fibrosis, is independently associated with new atrial arrhythmia in patients with pre-existing cardiac disease but without a prior history of atrial arrhythmia. Furthermore, LA LGE is associated with indices of worsening LV diastolic function. These findings are novel, as previous studies of atrial LGE have almost exclusively examined patients with pre-existing AF. In our study LA LGE had a stronger relationship to new-onset atrial arrhythmia than some previously reported predictors, including LA volume, LA function and hypertension. In a subset of patients with available data, LA LGE was independently associated with new-onset atrial arrhythmia when included in a multivariable logistic regression model with the CHARGE-AF risk score. The association between indices of atrial myocardial function and LA LGE support the concept that LA LGE represents fibrosis of the LA which likely arises as a consequence of mechanical and hemodynamic factors.

Atrial tachycardia was the most commonly developed arrhythmia in our study (9 of 15). It is an atrial rhythm at a rate of > 100 beats per minute, usually paroxysmal and self-limited, which originates outside of the sinus node. Multifocal atrial tachycardia may precede a diagnosis of AF [27–31]. Atrial arrhythmias complicating cardiac disease are associated with poor clinical outcomes.

New-onset AF in patients with existing heart failure is an independent predictor of in-hospital mortality and its treatment by ablation has recently been shown to improve outcome for patients with heart failure, although not for all patients [32, 33]. Novel biomarkers which could identify patients at risk of new atrial arrhythmia would therefore be helpful. Our study identified an LA LGE threshold of 10% of LA volume as strongly predicting new onset arrhythmia. Confirmation will require replication in future cohorts and clinical utility depends upon an effective therapeutic intervention (e.g. stroke or arrhythmia prevention). Existing risk models, for example the CHARGE-AF score, can be used to predict AF using routine clinical data without further testing. Such approaches are cheap and effective, and therefore novel and more expensive biomarkers such as LA LGE, must be shown to provide sufficiently added value in prospective studies.

The mechanisms leading to the development of atrial fibrosis are incompletely understood. Hemodynamic factors are one component of this process, and our data support the paradigm of LA structural changes arising as a consequence of diastolic impairment [34–37]. More extensive LA LGE was present in patients with worsening diastolic dysfunction (E/e’), larger atria and lower LA EF; but was unrelated to LV systolic function. LA volume is an increasingly used biomarker in clinical practice and has been associated with outcomes in patients with heart failure [38]. However, we have shown that LA LGE is not simply a surrogate for LA volume, given its superior and independent relationship with clinical outcomes in this study. The superior correlation of minimal compared with maximal LA volume with LA LGE is consistent with prior studies which have shown that minimum LA volume correlates better with AF development, adverse cardiovascular events, and N-terminal pro b-type natriuretic peptide (NT-proBNP) [39–41]. Overall, the finding that LA LGE is correlated with parameters of diastolic function, especially E/e’, a correlate of LA pressure, is novel and important.

Although there were a small number of subjects with hypertrophic cardiomyopathy, those subjects had substantially higher LA LGE percentages compared to other participants. To date, studies of LGE in hypertrophic cardiomyopoathy have primarily been limited to ventricular LGE. Such studies have shown ventricular LGE to be linked to AF development [42] and diastolic dysfunction [43, 44]. Our data is also consistent with a recent study by Cochet et al. who found a correlation of LA LGE with age and structural heart disease. [19] We also found that increasing age was associated with increased LA LGE, with strength of association similar to that found between age and peak LA strain in a recent study [45].

The presence of any LV LGE was not found to be correlated to atrial LGE in the study, showing that atrial LGE is not simply a cumbersome stand-in for LV scar. Furthermore, LV LGE was not correlated to new atrial arrhythmia in this study.

These data support the concept of LA LGE as a prognostic biomarker of the fibrotic and electrically susceptible atrial substrate. LA LGE was associated with some traditional risk factors for atrial arrhythmias, and particularly for atrial fibrosis [46], including age, larger LA volume, HCM, and diastolic dysfunction. LA LGE may have a unique role—beyond these risk factors—in identifying patients at risk of atrial arrhythmia.

## Limitations

There are several limitations to our study. This study is a retrospective analysis of patients with a range of cardiac diagnoses undergoing CMR myocardial assessment. Only 70% of the cohort had an echocardiogram within a year of CMR, and the median delay was 61 days, which limits the comparison of echocardiographic diastolic parameters with LA LGE. The presence of a relationship between atrial LGE and clinical outcome in such a broad group is a strength of the study, but is limited by a possible selection bias, arising from clinician referral for CMR. Importantly, almost all of our subjects were assessed in the context of pre-existing cardiac disease; we cannot therefore infer that atrial LGE is related to clinical outcome in the general population.

One of the major difficulties in any study of atrial arrhythmia is adequate followup and identification of affected individuals. In our definitions of the presence/absence of atrial arrhythmia we included subjects who reported no symptoms and had negative 12 lead ECG findings. It is possible that some of these patients experienced paroxysmal atrial arrhythmia which would have influenced our analysis. However, we performed a sensitivity analysis of patients in whom longer term monitoring was available and found similar and significant results. It is also possible that patients had subclinical atrial arrhythmia prior to study enrollment, and events in our study represent the transition to clinical manifestation. However, LA LGE performed better at identifying new onset (or transition to overt) arrhythmia than other previously described predictors including LA size and function.

Atrial arrhythmias can be misclassified by certain implantable devices, which use detection algorithms based on analysis of Lorenz plots for classification. Given our inclusion of such devices in our cohort, we cannot exclude the possibility that occasional misclassification could occur.

Although we found higher LA LGE in the patients who died, consistent with the recent findings of King et al. [7], death events were infrequent, and the cause of death could not be identified in all cases, thus limiting our statistical analysis.

LA LGE imaging and quantification has limitations. The relatively high rate of suboptimal scans (40%) found in the current study reflects a “learning curve” in use of the method at our institution. Scans were non-diagnostic due to imaging too early (< 15 min) or too late (> 30 min) post-injection, or poor TI choice. These were mainly preventable causes of poor quality, especially common early in protocol utilization at our center. The rate of non-diagnostic scans in the last year of the study was 19%, very similar to other reports of 17% [10, 19]. Less preventable sources of reduced image quality are poor respiratory compensation, and artifacts in patients with arrhythmia. Arrhythmia sometimes causes ghosting artifacts, poor nulling, and lower signal to noise ratio (SNR), as recently described [47].

Because atrial LGE images always demonstrate aortic and valvular enhancement, in the earliest studies there was concern that atrial enhancement was non-specific. However, valves and arterial walls are largely composed of extracellular matrix, including elastin and collagen, which explains these phenomena. CMR has a limited spatial resolution compared to the thickness of the atrial wall, and consequently there is a partial volume error associated with evaluation of LGE in the LA myocardium. The contrast in LA LGE is affected by the chosen TI and the imaging time post-injection, which leads to the necessity of patient specific thresholds for segmentation. TI choice, and therefore contrast and SNR, is also influenced by the presence of LV diffuse fibrosis [47]. Patient-specific thresholding based on valvular enhancement has limitations, since it is highly operator dependent. However, this segmentation method was shown to correlate robustly with expert consensus [23]. Furthermore, here we report good inter- and intra-observer reproducibility for LA LGE, similar to that of other studies [19]. In our study, the LA myocardial volume was approximated as a scalene ellipsoid, which may introduce a further error. For all of these reasons, acquisition and quantification of LA LGE is challenging and LA LGE represents only an estimate of LA fibrosis. Even with all of these concerns, a recent study on post-ablation atrial LGE showed excellent reproducibility in scar quantitation, with imaging sessions separated by two days [12].

## Conclusion

The extent of LA LGE is associated with new atrial arrhythmia in patients with pre-existing cardiac disease. LA LGE had a stronger relationship than previously reported predictors, including LA volume and function. There is also a moderate relationship between increasing LA LGE and increasing LA volume, decreasing LA function and worsening LV diastolic function. These data suggest LA LGE may be useful as a prognostic biomarker of new onset atrial arrhythmia through characterization of the fibrotic and electrically susceptible atrial substrate. Further investigation to develop possible clinical applications is warranted.

## Additional file

Additional file 1: Inter- and Intra-observer variability for atrial LGE measurement. (DOCX 1303 kb)

## Abbreviations

AF: atrial fibrillation
AUC: area under the curve
BSA: body surface area
CI: confidence interval
CMR: cardiovascular magnetic resonance
CNR: contrast to noise ratio
ECG: electrocardiogram
EF: ejection fraction
ICC: intraclass correlation
ICD: implanted cardiodefibrillator
IQR: interquartile range
LA: left atrium/left atrial
LGE: late gadolinium enhancement
LV: left ventricle/left ventricular
NT-proBNP: N-terminal pro b-type natriuretic peptide
OR: odds ratio
PPM: permanent pacemaker
SNR: signal to noise ratio
TE: echo time
TR: repetition time
