# Supplementary material for: Prognostic and functional implications of left atrial late gadolinium enhancement cardiovascular magnetic resonance
Source: J Cardiovasc Magn Reson. 2019 Jan 3;21:2. doi: 10.1186/s12968-018-0514-3 (PMC6317232; doi:10.1186/s12968-018-0514-3)
Supplement: Supplementary file 1 — Inter- and Intra-observer variability for atrial LGE measurement. (DOCX 1303 kb) [file 12968_2018_514_MOESM1_ESM.docx]

**Additional file 1**

***Inter- and Intra-observer Variability***

**Methods**

The last 26 consecutive datasets included in the study were selected for analysis for inter and intra-observer variability. They were first graded for image quality and myocardial nulling (1=poor, 2=fair, 3=good). Two were excluded prior to the analysis of inter- and intra-observer variability because the images, although they had fair quality, exhibited poor nulling. Images were post-processed by Observer A (with >10 years’ experience analyzing atrial LGE) on two occasions separated by one week, and once by Observer B (no prior experience in atrial LGE segmentation), both blinded to any other measurements. Observers were chosen to provide an estimate of best (expert segmentation) and worst-case (novice segmentation) LA LGE segmentation. Before the analysis, they agreed upon the segmentation method, as described below.

3D Slicer software (<http://www.slicer.org>, NA-MIC) was used to define thresholds and segment LA LGE. Thresholds were set using mitral/aortic valve enhancement as the reference for each image, while attempting to exclude blood pool enhancement and include visually apparent LGE (Supplemental Figure 1A). LA LGE was then segmented using this threshold and a 2mm “paint brush” tool, applied to regions with visually apparent LGE, avoiding artifacts, throughout the whole atrial volume. LGE in the pulmonary vein sleeves were segmented out to 5mm beyond the ostia. The volume of LGE was expressed as %LGE, by normalizing by the volume of atrial myocardium, as described in Methods. The Bland-Altman limits of agreement and the intraclass correlation coefficient (ICC) were calculated.

**Results**

***Inter- and Intra-observer Variability***

Average segmentation time was 5 ± 2 minutes for observer A. One subject was as an extreme outlier, due to observer B excluding an area of high signal intensity considered to be LGE (see outlier in Supplemental Figure 2A). Results are reported both with and without this outlier. The mean volume of LGE was 0.6 ± 0.6ml, and mean LGE % was 2.7 ± 2.8 % of the LA myocardial volume. Analysis of the 24 subjects revealed good inter and intra-observer variability (Supplemental Table 1) and shown in Supplemental Figure 2, with inter-observer ICCs of 0.71 (moderate) to 0.82 (moderate/good, excluding the outlier), and intra-observer ICC of 0.94 (excellent).

| % LGE | **Bias ±2SD** | R | ICC |
| --- | --- | --- | --- |
| Inter-observer | 0.6% ± 7.3% | 0.73 p=0.002 | 0.71 |
| Inter-observer* | 1.1% ± 5.1% | 0.90, p<0.001 | 0.82 |
| Intra-observer | -0.3% ± 2.9% | 0.94, p<0.001 | 0.94 |

Table S1: Inter-observer and intra-observer variability

*excluding the outlier data point.

Figure S1: Segmentation example. The left panel shows the initial threshold choice, which includes the enhanced valves, and excludes blood signal. Panels 2-4 show segmentation by Observer A (two times) and Observer B. During segmentation, care is taken to avoid aortic wall, mitral valvular apparatus, or artifacts as LGE enhancement.


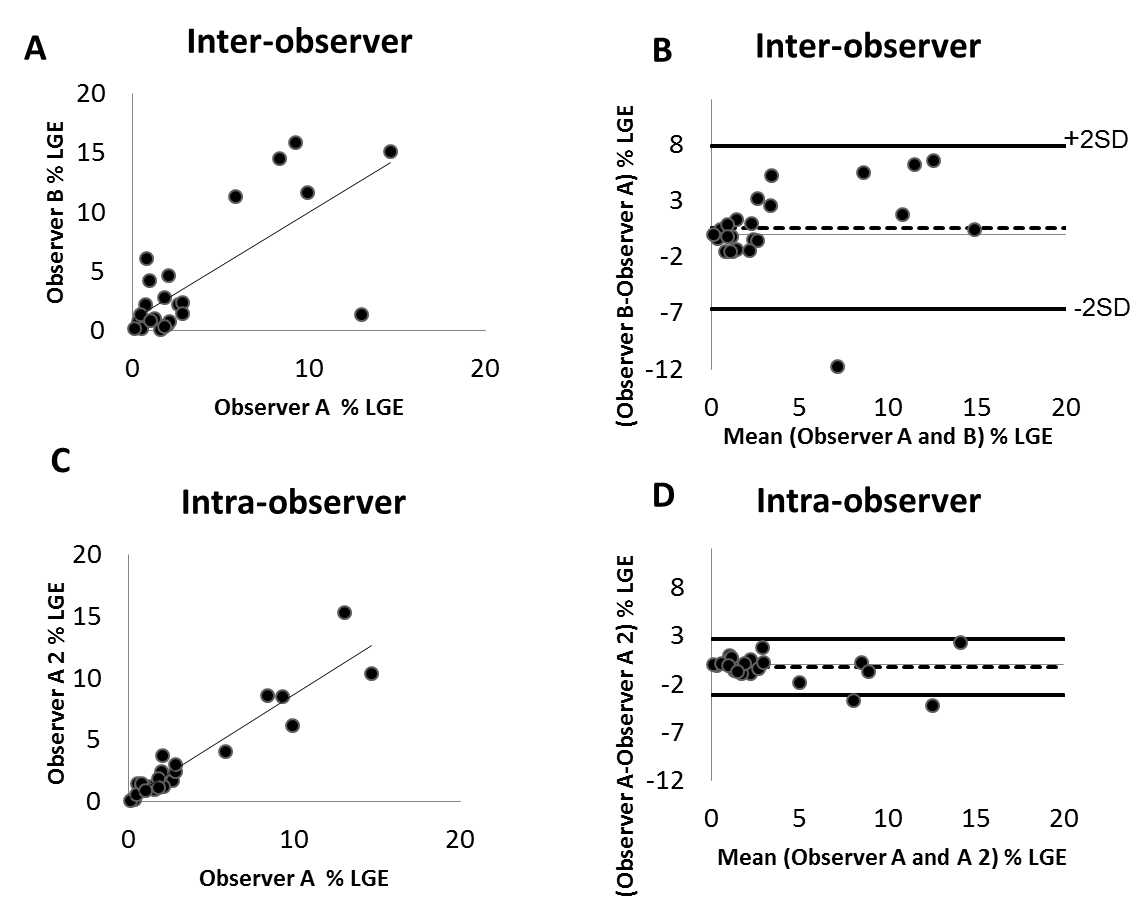


Figure S2: Inter-observer variability (A,B) and intra-observer variability (C,D), showing good agreement. The correlations are shown in (A,C) with Bland-Altman plots in (B,D), showing the bias and ±2SDs. Note the single outlier is shown in (A). The variability was calculated with and without this outlier, in Table 1; the results reported in the main study include the outlier.
